# National Institutes of Health funding to neurosurgery departments, FY2015–FY2025: real-terms trends, training infrastructure, and MeSH retrieval coverage

**DOI:** 10.1016/j.bas.2026.107486

**Published:** 2026-10-24

**Authors:** Aaron Lawson McLean, Julian Kahr, Christian Senft

**Affiliations:** Department of Neurosurgery, Jena University Hospital, Friedrich Schiller University Jena, Jena, Germany

**Keywords:** Neurosurgery, NIH funding, Research funding, Surgeon-scientist, Bibliometrics, Medical subject Headings, Health workforce

## Abstract

**Introduction:**

Federal research funding and publication output are among the principal quantitative measures of academic neurosurgery, and the validity of each depends on how it is measured. We characterised National Institutes of Health (NIH) funding to neurosurgery departments in real terms, examined whether early-career award trajectories can be partially characterised from public administrative data, and quantified the coverage of MeSH-based publication retrieval.

**Methods:**

We analysed all NIH Research Portfolio Online Reporting Tools (RePORTER) grants administratively assigned to “NEUROSURGERY” departments (FY1992–FY2026; accessed 8 March 2026), deduplicated to 8480 grant-year records across 87 institutions. Funding was deflated to constant 2025 US dollars using the Consumer Price Index. Institutional concentration was measured with the Gini coefficient, the Herfindahl–Hirschman Index, and top-share ratios. We examined transitions from early-career (fellowship or K) to independent (R-type) awards, modelled the association between institutional T32 training grants and later R01-investigator counts with adjustment for baseline research intensity, tested the recent funding trajectory against its prior trend, and quantified MeSH retrieval coverage of neurosurgical publications via NCBI E-utilities.

**Results:**

Real funding to neurosurgery departments rose from $131.2M (FY2015) to a peak of $268.5M (FY2023; real compound annual growth 6.5%), then declined to $245.7M (FY2025); the FY2024 and FY2025 values were both lower than expected under the extrapolated FY2015–FY2023 trend. The peak and subsequent decline were unchanged by deduplication. From FY2023 to FY2025 the departmental series fell 8.5%, compared with a 1.1% real decline in NIH-wide research project grant obligations and a 5.7% decline in NIH-wide award counts. R01 and R37 awards provided 56.2% of funding, and institutional concentration was stable (Gini 0.54–0.58). Within department-attributed data, later independent R-type funding was much more frequent after mentored K awards (40.4%) than after fellowships (8.0%). Departments holding a T32 grant had more distinct R01 investigators (unadjusted incidence rate ratio 3.5), but this association did not persist after adjustment for baseline departmental funding and prior R01-investigator count (adjusted incidence rate ratio 1.07; 95% CI 0.45–2.56). MeSH-restricted retrieval returned less than half as many neurosurgical records as keyword-augmented retrieval by 2023.

**Conclusions:**

Departmental NIH funding grew in real terms but has fallen below its prior trend since FY2023. Mentored K awards, more than fellowships, preceded independent funding, whereas the apparent T32 advantage reflected pre-existing departmental research intensity. The wider NIH benchmark suggests a shared post-2023 funding constraint, but the departmental decline was steeper. MeSH-restricted retrieval substantially under-captures the neurosurgical literature relative to broader keyword-augmented and journal-anchored strategies. Funding and bibliometric indicators of academic neurosurgery should both be read with attention to measurement validity; all results describe departments as administrative units, not individual neurosurgeons.

## Highlights

• Real NIH funding to neurosurgery departments grew despite a post-FY2023 decline.
• The post-FY2023 decline was steeper than the NIH-wide RPG funding change.
• Independent R funding more often followed mentored K awards than fellowships.
• A T32 department's larger R01 portfolio reflected pre-existing research intensity.
• MeSH-restricted search returned under half the records of keyword-augmented search.

## 1 Introduction

Federal research funding is a structural determinant of academic neurosurgery, shaping protected research time, training capacity, and the scientific output of the specialty. The National Institutes of Health (NIH) is the dominant public sponsor of biomedical research in the United States, and its support of neurosurgery departments has been examined before. ReFaey and colleagues characterised NIH funding to neurosurgeon-scientists from 1993 to 2017 and, using manual investigator-level adjudication, reported that the proportion of awards held by neurosurgeon-scientists declined even as aggregate departmental funding rose (ReFaey et al., 2021). Hauptman and colleagues documented parallel trends in neurosurgical publication output (Hauptman et al., 2011). More recent subspecialty work has raised concern about the surgeon-scientist pipeline, including a reported contraction among cerebrovascular surgeon-scientists (Pugazenthi et al., 2024; Nguyen et al., 2023). Cross-specialty comparisons consistently place neurosurgery among the more modestly funded surgical disciplines, although estimates depend on whether funding is attributed to departments or to individuals (Schlafly and Sebro, 2022; Imam et al., 2023; Saif et al., 2022).

Three questions are not resolved by this literature. First, prior descriptive series have generally reported nominal dollars; because input costs rise over time, nominal growth overstates the real expansion of purchasing power (Moses et al., 2005; Lauer et al., 2023). Second, prior work has been largely descriptive and has not tested mechanistic hypotheses — for example, whether early-career training awards are followed by independent funding, or whether institutional training infrastructure is associated with later research capacity. Third, the funding environment has shifted materially since 2023, so an analysis whose window ends in 2017 cannot speak to the current trajectory.

Measurement of academic neurosurgery also depends on bibliometric retrieval methods, because publication output is commonly used alongside funding as an indicator of departmental research performance (Hauptman et al., 2011). Bibliometric assessment frequently relies on retrieving publications indexed with Medical Subject Headings (MeSH) (Hauptman et al., 2011; Kamil et al., 2023). MeSH terms are assigned after publication, and the National Library of Medicine (NLM) transitioned MEDLINE indexing to an automated algorithm in 2022 (National Library of Medicine, 2021; Chen et al., 2023). If MeSH-based retrieval captures a shrinking fraction of the relevant literature, comparisons of research output across time will be biased (Irwin and Rackham, 2017; Hadfield, 2020).

These two problems — how funding is attributed to departments, and how publications are retrieved for bibliometric assessment — share a common theme: the validity of the routine measurements used to evaluate academic neurosurgery. We therefore undertook a reproducible analysis of NIH funding to neurosurgery departments with four aims: (i) to quantify funding in inflation-adjusted terms and test whether the recent trajectory departs from trend; (ii) to characterise institutional concentration with interpretable inequality metrics; (iii) to examine, within public administrative data, whether early-career awardees later hold independent funding and whether institutional research training grants (T32) are associated with later investigator-initiated research project grant (R01) capacity once baseline research intensity is taken into account; and (iv) to quantify MeSH retrieval coverage of the neurosurgical literature. Throughout, the analysis concerns funding to neurosurgery departments as administrative units and not to neurosurgeons as individuals; this distinction is developed in the Discussion.

## 2 Materials and methods

### 2.1 Study design and data source

This was a longitudinal analysis of administrative grant data, reported in accordance with applicable items of the RECORD extension to the STROBE statement for studies using routinely collected data. The primary data source was the NIH RePORTER database (https://reporter.nih.gov), a publicly available record of NIH-funded projects. The database was queried through the RePORTER web interface on 8 March 2026. The query set the Department field to “NEUROSURGERY”, with no restriction on fiscal year, activity code, or funding institute, and the matching records were exported. The Department field reflects organisation-supplied metadata; records filed under variant labels (for example, “NEUROLOGICAL SURGERY” or abbreviated forms) or with a missing Department value are, by design of this exact-string filter, not part of the cohort. This is a recognised constraint of department-level RePORTER queries and is revisited in the Discussion; the same departmental approach has been used in funding analyses of other surgical specialties (Imam et al., 2023).

### 2.2 Cohort definition and deduplication

RePORTER exports can contain duplicate application records. We identified 164 Application IDs that appeared on more than one row and removed surplus records, retaining for each Application ID the row with the largest reported Total Cost. Because RePORTER assigns one Application ID per application, rows sharing an Application ID are repeated exports of the same award record rather than distinct subprojects or supplements. We prespecified a sensitivity comparison of annual funding before and after deduplication. Because RePORTER coverage is sparse before the mid-1990s and the comparator literature uses recent windows (ReFaey et al., 2021; Imam et al., 2023), the primary analysis window was FY2015–FY2025; FY2026 was excluded as incomplete at the query date. The cohort captures all grants administratively housed in departments labelled “NEUROSURGERY”, irrespective of the degree or specialty of the PI.

### 2.3 Inflation adjustment and budget context

Nominal award amounts (RePORTER “Total Cost”) were deflated to constant 2025 US dollars using annual-average values of the Consumer Price Index for All Urban Consumers (CPI-U; Bureau of Labor Statistics series CUUR0000SA0). CPI-U was chosen because it is a transparent, publicly verifiable index that supports exact reproduction; it is a general measure of purchasing power rather than a biomedical input-cost index, and is interpreted here as a real-dollar adjustment, not as an adjustment for the specific cost of conducting research (Lauer et al., 2023). The 2025 annual average (321.943) is the value published by the Bureau, computed from eleven monthly observations because the October 2025 observation was not collected during the federal shutdown. Compound annual growth rates were computed for the primary window. For a system-wide benchmark, we obtained annual NIH-wide research project grant (RPG) obligations and award counts from the NIH Data Book (https://report.nih.gov/nihdatabook/category/4), deflated the obligation series with the same CPI-U values, and compared FY2023–FY2025 changes. Departmental funding was also expressed as an approximate share of the total NIH appropriation. These denominators have broader scope than the department-filtered numerator and are interpreted as contextual, not like-for-like, comparisons.

### 2.4 Institutional concentration

For each fiscal year, institutional funding was summed by Organization Name. Each grant-year was attributed to the organization recorded for that award year, so an investigator who transferred institutions contributed to the former and new institution in the corresponding years. PI-level trajectories instead followed the stable Contact PI Person ID across organizations. Concentration was summarised with the cumulative shares of the five and ten largest recipients, the Herfindahl–Hirschman Index (HHI; the sum of squared percentage shares, ranging from near 0 for an atomistic distribution to 10,000 for a single recipient, with values below 1500 conventionally regarded as unconcentrated), and the Gini coefficient (ranging from 0 when every department is funded equally to 1 when one department holds all funding). Top-share ratios and the HHI are the primary concentration measures; the Gini coefficient is reported as a continuous summary (Aagaard et al., 2020).

### 2.5 Disease-domain classification

Each grant was screened for seven disease domains (neuro-oncology, cerebrovascular, spine, functional/epilepsy, trauma/traumatic brain injury, hydrocephalus, and peripheral nerve) by case-insensitive matching of a keyword lexicon against concatenated text fields. These domains are not symmetrical categories — some are diseases, some anatomical or clinical areas — and the resulting amounts are therefore described as keyword-attributed disease-domain funding rather than as definitive subspecialty totals. Multi-word terms were matched as substrings; short abbreviations were matched with word-boundary constraints. Grants matching several domains contributed to each (multi-label); grants matching none were classed as Other/Unclassified. Because keyword screening is imprecise, rank stability was verified against an independent classification using only the project title and NIH Spending Categorization. The full lexicon is given in the Supplementary Material.

### 2.6 Early-career award trajectories

To examine whether early-career awardees later held independent funding, PIs were tracked by RePORTER Contact PI Person ID. RePORTER provides a Person ID only for the contact PI; additional investigators on multiple-PI awards appear as names without identifiers, so this analysis captures administrative leadership as contact PI and does not capture non-contact multiple-PI roles (14.7% of grant-years in the cohort listed more than one PI). An early-career-entry cohort was defined as PIs whose first award in the cohort was a fellowship (F30/F31/F32) or a mentored career-development award (K-series); the primary cohort was restricted to first awards in FY2019 or earlier, providing at least six fiscal years of potential follow-up through FY2025, which exceeded the observed median five-year interval among converters. We repeated the calculation with FY2017, FY2018, and FY2020 cutoffs. The outcome was subsequently appearing as contact PI on an independent research award (R01, R37, RF1, R35, or DP). Fellowships and K awards differ in career stage and purpose — K awards are explicitly designed to bridge to independent funding whereas fellowships predominantly support earlier training — so the comparison characterises observed trajectories and is not an evaluation of mechanism effectiveness. This is an administrative trace within the RePORTER department-defined universe, not a complete career pipeline.

To test whether the department-based cohort was dominated by neurosurgeons, we manually audited the 20 contact PIs with the greatest cumulative nominal funding during FY2015–FY2025. PI type (neurosurgeon, non-neurosurgeon physician, or non-physician scientist) was assigned from official institutional biographies; the classifications and source URLs are reported in the Supplementary Material.

### 2.7 Institutional T32 association and sustainability

At the institutional level, we examined whether holding a T32 institutional training grant during FY2010–FY2020 was associated with the count of distinct R01 contact PIs in FY2021–FY2025. Because T32-holding departments are, a priori, the larger and more research-intensive departments, the association was estimated both unadjusted and in a negative-binomial regression adjusted for baseline (FY2010–FY2020) total NIH funding (log-transformed) and the baseline count of distinct R01 PIs. Results are reported as incidence rate ratios (IRRs). Institutional sustainability was summarised by year-over-year retention (the fraction of departments funded in year Y also funded in Y+1) and by each institution's first funded year.

### 2.8 Trend testing and MeSH retrieval coverage

Real funding was tested for monotonic trend with the Mann–Kendall test. To assess whether the post-2023 trajectory departed from trend, a log-linear ordinary least-squares model was fitted to FY2015–FY2023 (nine points) and 95% prediction intervals were computed for FY2024 and FY2025 (two points). Because the data are an administrative census rather than a probability sample, these intervals are used descriptively, to indicate departure from the extrapolated trend rather than sampling improbability. MeSH retrieval coverage of the neurosurgical literature was quantified by querying NCBI E-utilities on 20 May 2026. For each publication year 2015–2025 we computed (i) a general-PubMed ratio of a MeSH-restricted query to a keyword-augmented query, and (ii) a journal-anchored ratio of MeSH-indexed to all articles in nine core neurosurgery journals. The keyword-augmented and journal-anchored queries are reference sets for comparison, not gold standards. Full search strings are in the Supplementary Material; ratios for 2024–2025 are reported but excluded from trend testing because indexing of recent literature is incomplete.

### 2.9 Statistical analysis, reproducibility, and ethics

Analyses used Python 3.12 (pandas, NumPy, SciPy, statsmodels). Two-sided p < 0.05 was treated as the threshold for statistical significance where inferential tests were applied. Analysis code, aggregate outputs, deflator values, and provenance instructions are openly available (see Declarations). The study used publicly available administrative data and aggregate bibliometric counts, did not constitute human-subjects research, and did not require institutional review board approval.

## 3 Results

### 3.1 Cohort

The export contained 8672 rows. Of 164 Application IDs that occurred more than once, all repeated copies carried identical Total Cost values; removing 192 surplus rows reduced reported funding by $134.9M (3.9%). The deduplicated cohort comprised 8480 grant-year records (8411 unique projects) at 87 institutions, with 1119 distinct contact PIs and total funding of $3.32 billion across FY1992–FY2026.

### 3.2 Funding trends and the recent inflection

In constant 2025 dollars, annual funding to neurosurgery departments rose from $131.2M in FY2015 to a peak of $268.5M in FY2023, then declined for two consecutive years to $255.3M (FY2024) and $245.7M (FY2025) (Fig. 1). The real compound annual growth rate over FY2015–FY2025 was 6.5%, against a nominal rate of 9.8%; approximately one-third of nominal growth therefore reflects inflation rather than expanded purchasing power. Real funding rose monotonically through FY2023 (Mann–Kendall τ = 0.94 for FY2015–FY2023) and the trend over the full window remained positive (τ = 0.82). However, both the FY2024 and the FY2025 values were lower than expected under the log-linear trend extrapolated from FY2015–FY2023, falling below its 95% prediction interval (FY2024 observed $255.3M versus projected $302.1M [interval $271.2–336.6M]; FY2025 observed $245.7M versus projected $329.9M [$294.3–369.8M]). The two-year decline of 8.5% from the FY2023 peak is therefore not consistent with simple extrapolation of the prior trend, although two post-peak observations cannot establish a durable reversal.

**Fig. 1:**
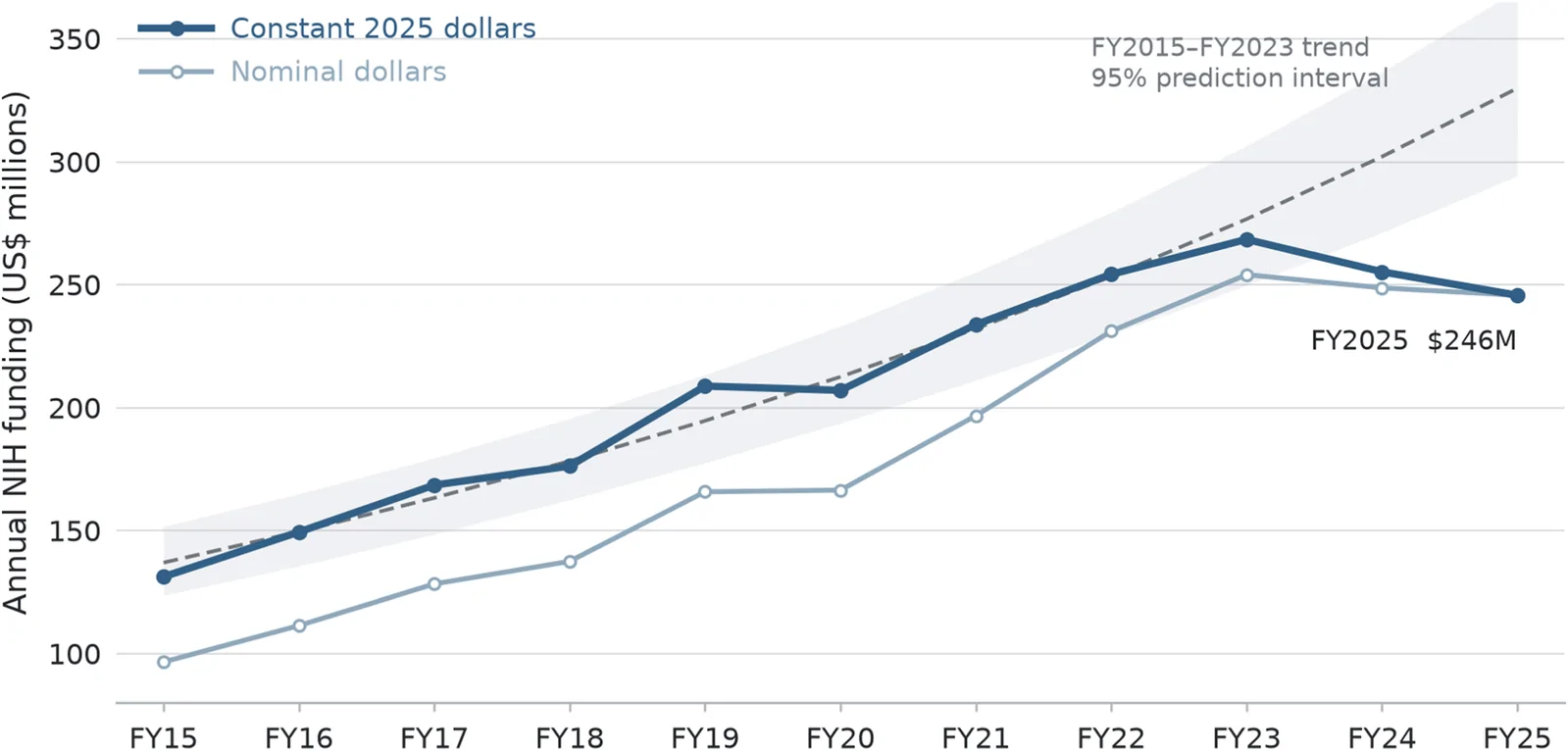
Annual NIH funding to neurosurgery departments, FY2015–FY2025, in nominal and constant 2025 US dollars (CPI-U deflated). The dashed line and shaded band show a log-linear trend fitted to FY2015–FY2023 real funding and its 95% prediction interval; the fit and interval are computed on the logarithmic scale and back-transformed to dollars for display. FY2024 and FY2025 fall below the interval. Source: NIH RePORTER, accessed 8 March 2026.

This pattern was not an artefact of deduplication: in the undeduplicated export the real-funding series also peaked in FY2023 and declined by 8.1% to FY2025 (versus 8.5% after deduplication), and the peak fiscal year was unchanged (Supplementary Fig. S1). From FY2023 to FY2025, the real departmental series fell 8.5%; over the same interval, NIH-wide RPG obligations fell 1.1% in real terms, the total NIH appropriation fell 6.5% in real terms, and the NIH-wide RPG award count fell 5.7% (Supplementary Fig. S2). The common direction argues against a wholly neurosurgery-specific explanation, but the departmental decline was steeper than the broader RPG funding change. Departmental funding expressed as a share of the total NIH appropriation rose from approximately 0.3% (FY2015) to 0.5% (FY2023–FY2025); this approximate benchmark is shown in the Supplementary Material and is not interpreted as an exact extramural share.

### 3.3 Funding mechanisms

R01 and R37 awards together provided 56.2% of real funding over FY2015–FY2025 (R01 alone, 54.5%; Table 2). Cooperative agreements (U01, 8.4%), specialized centre grants (P50, 5.8%), and exploratory awards (R21, 4.6%) followed. The combined share of programme and centre grants (P01, P30, P50) fell from 10.8% of funding in FY2015 to 5.4% in FY2025.

**Table 1:** The ten institutions receiving the most cumulative NIH funding to their neurosurgery departments, FY2015–FY2025 (constant 2025 US dollars). Source: NIH RePORTER, accessed 8 March 2026.

Rank	Institution	Grant-years	Cumulative real funding ($M)	Share of FY2015–FY2025 total (%)	FY2025 funding ($M)
1	University of California, San Francisco	489	302.6	13.2	28.6
2	Stanford University	345	186.0	8.1	12.7
3	Duke University	182	110.3	4.8	12.2
4	University of California, Los Angeles	155	104.8	4.6	7.0
5	University of Florida	143	104.0	4.5	21.8
6	Northwestern University	173	89.7	3.9	7.5
7	Washington University in St. Louis	174	88.8	3.9	14.9
8	University of Pennsylvania	143	83.3	3.6	14.9
9	Baylor College of Medicine	155	81.3	3.5	8.9
10	Yale University	172	79.0	3.4	4.0

Cohort total real funding over FY2015–FY2025 was $2.30 billion. Institution names follow NIH RePORTER organisation records.

**Table 2:** Funding mechanism mix, FY2015–FY2025 (constant 2025 US dollars). The twelve mechanisms with the largest cumulative real funding are listed individually; all remaining mechanisms are aggregated as “Other mechanisms”. Source: NIH RePORTER, accessed 8 March 2026.

Activity code	Mechanism	Real funding ($M)	Share (%)	Grant-years
R01	Research Project Grant	1253.1	54.5	2434
U01	Research Project Cooperative Agreement	193.5	8.4	158
P50	Specialized Center Grant	133.2	5.8	66
R21	Exploratory/Developmental Research Grant	106.5	4.6	360
UH3	Exploratory/Developmental Cooperative Agreement (phase II)	76.9	3.3	88
RF1	Multi-Year Funded Research Project Grant	66.6	2.9	32
P01	Research Program Project Grant	52.4	2.3	29
R35	Outstanding Investigator Award	46.0	2.0	49
UG3	Exploratory/Developmental Cooperative Agreement (phase I)	42.9	1.9	39
R37	Method to Extend Research in Time (MERIT) Award	39.5	1.7	72
T32	Institutional National Research Service Award	31.4	1.4	61
K08	Mentored Clinical Scientist Career Development Award	23.4	1.0	110
—	Other mechanisms (combined)	233.7	10.2	785

### 3.4 Institutional concentration

Across FY2015–FY2025 the top-ten share of departmental funding ranged from 53.5% to 61.3% with no secular trend, the HHI ranged from 408 to 651 — within the conventionally unconcentrated range — and the Gini coefficient ranged narrowly from 0.543 to 0.576 (Fig. 2A). In FY2025 the ten largest recipients held 56.3% of funding and median per-department funding was $2.75M (interquartile range $1.14M–$5.87M). The five largest cumulative recipients over the window were the University of California, San Francisco ($302.6M), Stanford University ($186.0M), Duke University ($110.3M), the University of California, Los Angeles ($104.8M), and the University of Florida ($104.0M) (Table 1). Although total funding more than doubled in real terms, its distribution across departments did not become measurably more or less concentrated; the FY2025 distribution is shown as a Lorenz curve in Supplementary Fig. S3. Forty-three of 707 contact PIs observed during FY2015–FY2025 were linked to more than one institution. Their PI-level histories were joined by Person ID, whereas institutional concentration assigned each grant-year to its recorded organization.

**Fig. 2:**
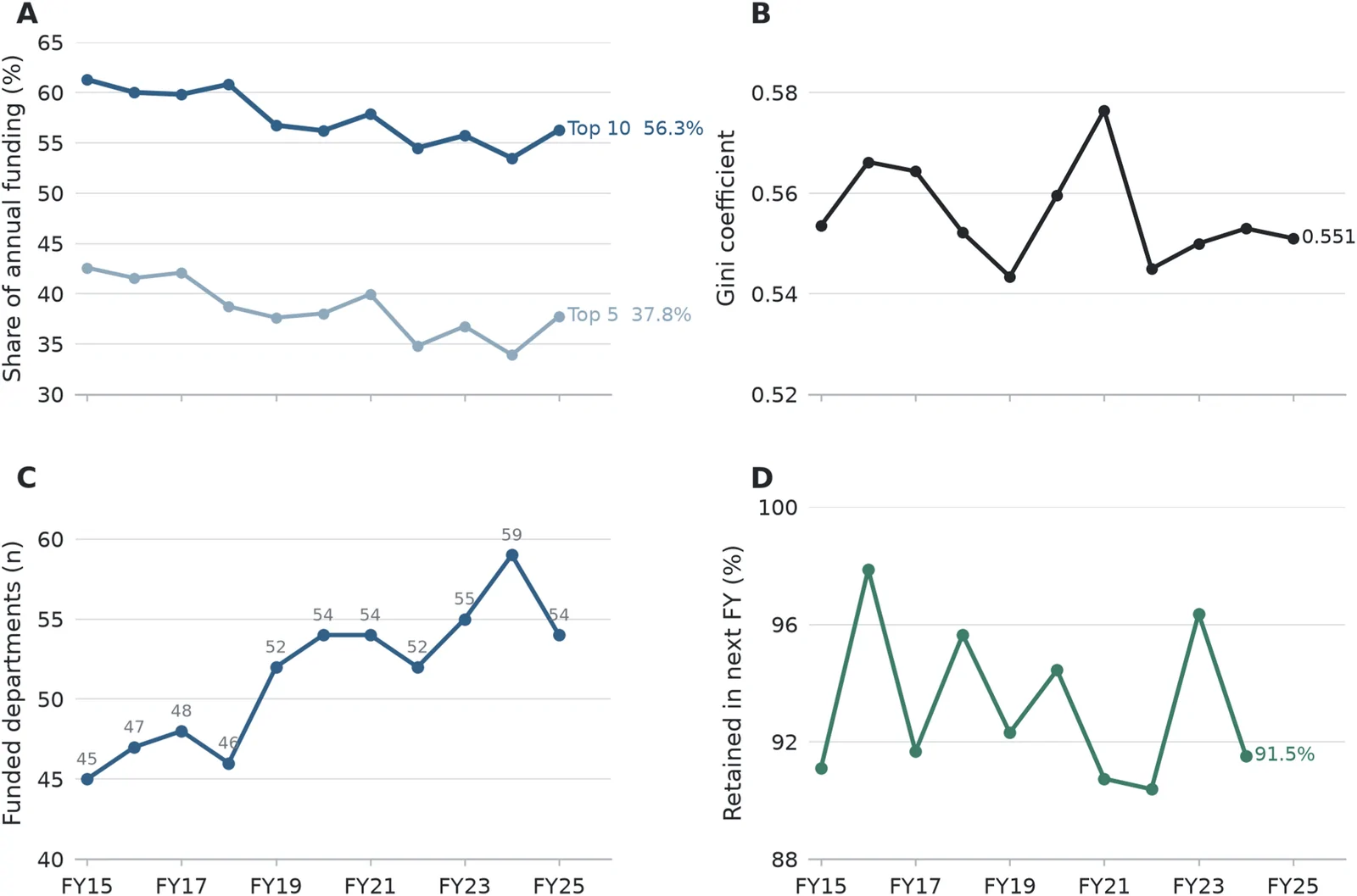
Institutional concentration of departmental funding, FY2015–FY2025. Aligned small multiples show (A) cumulative funding shares of the five and ten largest recipients, (B) the Gini coefficient, (C) the number of funded departments, and (D) year-over-year retention, defined as the percentage of departments funded in a year also funded in the next. Source: NIH RePORTER, accessed 8 March 2026.

### 3.5 Institutional sustainability

The number of departments funded in a given year rose from 45 (FY2015) to 59 (FY2024) before falling to 54 (FY2025) (Fig. 2B). This count growth reflected fuller participation of an established set of institutions rather than the entry of new ones: 73 of the 87 institutions had first received funding on or before FY2010, and only 7 were first funded during FY2015–FY2021. Year-over-year retention was high throughout (mean 93.2%). The FY2024-to-FY2025 contraction comprised five departments that lost funding and none that gained it; four of the five had been funded for 16 years or longer, and each had held only a single small grant in FY2024 (FY2024 amounts $0.05M–$0.51M), consistent with marginal departments losing a single award rather than structural attrition. An anonymised description of these five departments is given in the Supplementary Material.

### 3.6 Early-career award trajectories

Of 266 PIs who entered the cohort with a fellowship or mentored career-development award on or before FY2019, 55 (20.7%) subsequently held an independent research award, after a median of 5 years (interquartile range 4–9). Within department-attributed NIH data, later independent R-type funding was much more frequent after mentored K awards than after fellowships: 42 of 104 mentored-K entrants (40.4%) versus 13 of 162 fellowship entrants (8.0%) (Fig. 3A). Because K awards and fellowships occupy different career stages and serve different purposes, this difference is expected and characterises observed trajectories rather than the relative effectiveness of the two mechanisms. The transition estimate was stable across entry-year cutoffs: 21.9% for FY2017 or earlier, 20.9% for FY2018 or earlier, 20.7% for FY2019 or earlier, and 20.0% for FY2020 or earlier. In the focused audit of the 20 highest-funded contact PIs, 15 were neurosurgeons, 3 were non-neurosurgeon physicians, and 2 were non-physician scientists.

**Fig. 3:**
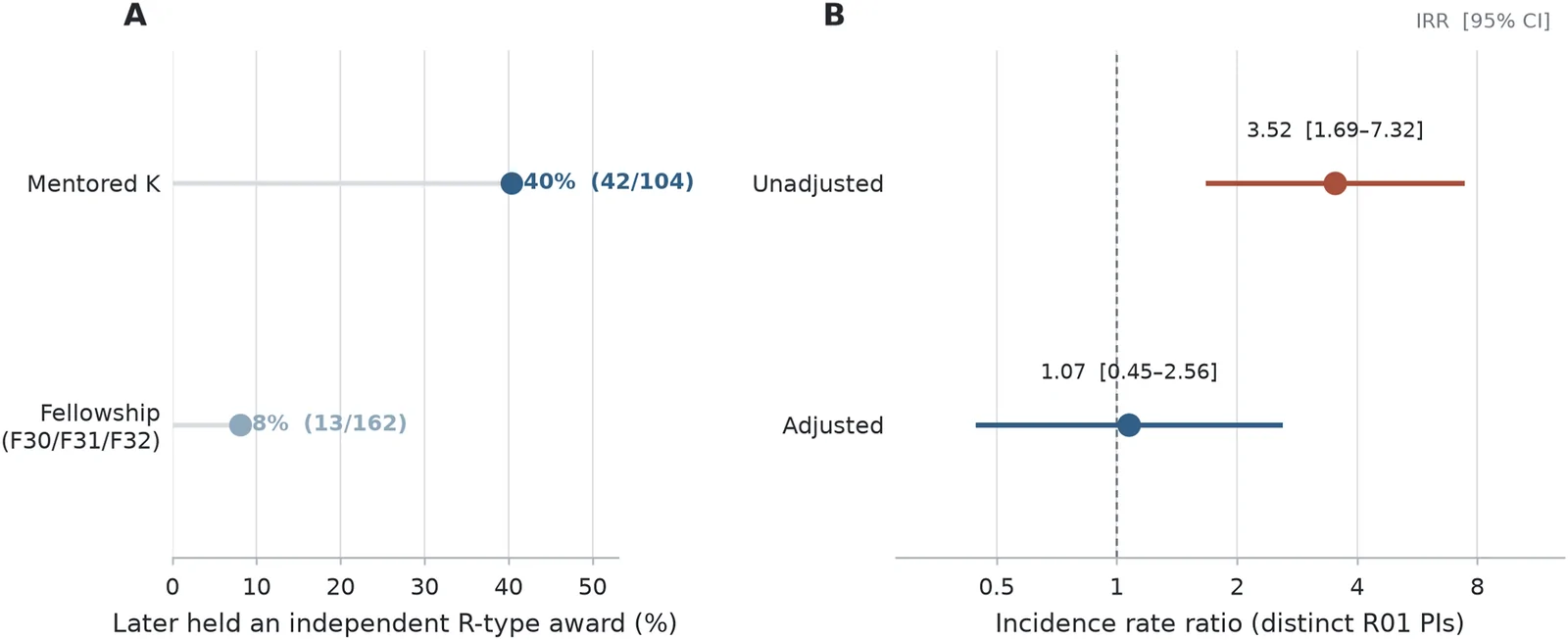
Early-career award trajectories and training infrastructure. (A) Percentage of early-career entrants (first cohort award in or before FY2019) who later held an independent R-type award, by entry mechanism; numerators and denominators are shown beside each estimate. (B) Incidence rate ratio for the count of distinct R01 contact PIs (FY2021–FY2025) in departments that held a T32 training grant (FY2010–FY2020), unadjusted and adjusted for baseline FY2010–FY2020 total funding and baseline R01-PI count (negative-binomial model); whiskers are 95% confidence intervals. Source: NIH RePORTER, accessed 8 March 2026.

### 3.7 Institutional T32 grants and R01 capacity

Departments that held a T32 training grant during FY2010–FY2020 (n = 9) had a median of 9 distinct R01 contact PIs in FY2021–FY2025, versus 1 for the 78 departments without a T32, corresponding to an unadjusted incidence rate ratio of 3.5 (Fig. 3B). This association did not persist after adjustment: in a negative-binomial model controlling for baseline (FY2010–FY2020) total NIH funding and the baseline count of R01 PIs, the adjusted incidence rate ratio for T32 presence was 1.07 (95% CI 0.45–2.56). The unadjusted association therefore reflects the fact that T32-holding departments were already the larger and more research-intensive departments, rather than an independent contribution of the training grant itself.

### 3.8 Disease-domain funding

Cumulative keyword-attributed real funding over FY2015–FY2025 was greatest for the neuro-oncology domain ($684M), followed by spine ($588M), cerebrovascular ($565M), functional/epilepsy ($491M), trauma/traumatic brain injury ($247M), hydrocephalus ($178M), and peripheral nerve ($95M) (Fig. 4); 14.6% of grants matched no domain. The leading position of neuro-oncology is consistent with the documented prominence of neurosurgery departments in highly cited glioblastoma research (Tazhibi et al., 2025). The rank order was identical under the independent title-plus-spending-category classification (Spearman ρ = 1.0), although absolute amounts were lower. These figures should be read as approximate, keyword-attributed amounts; differences among the closely ranked spine, cerebrovascular, and functional/epilepsy domains lie within the uncertainty of keyword screening and should not be over-interpreted.

**Fig. 4:**
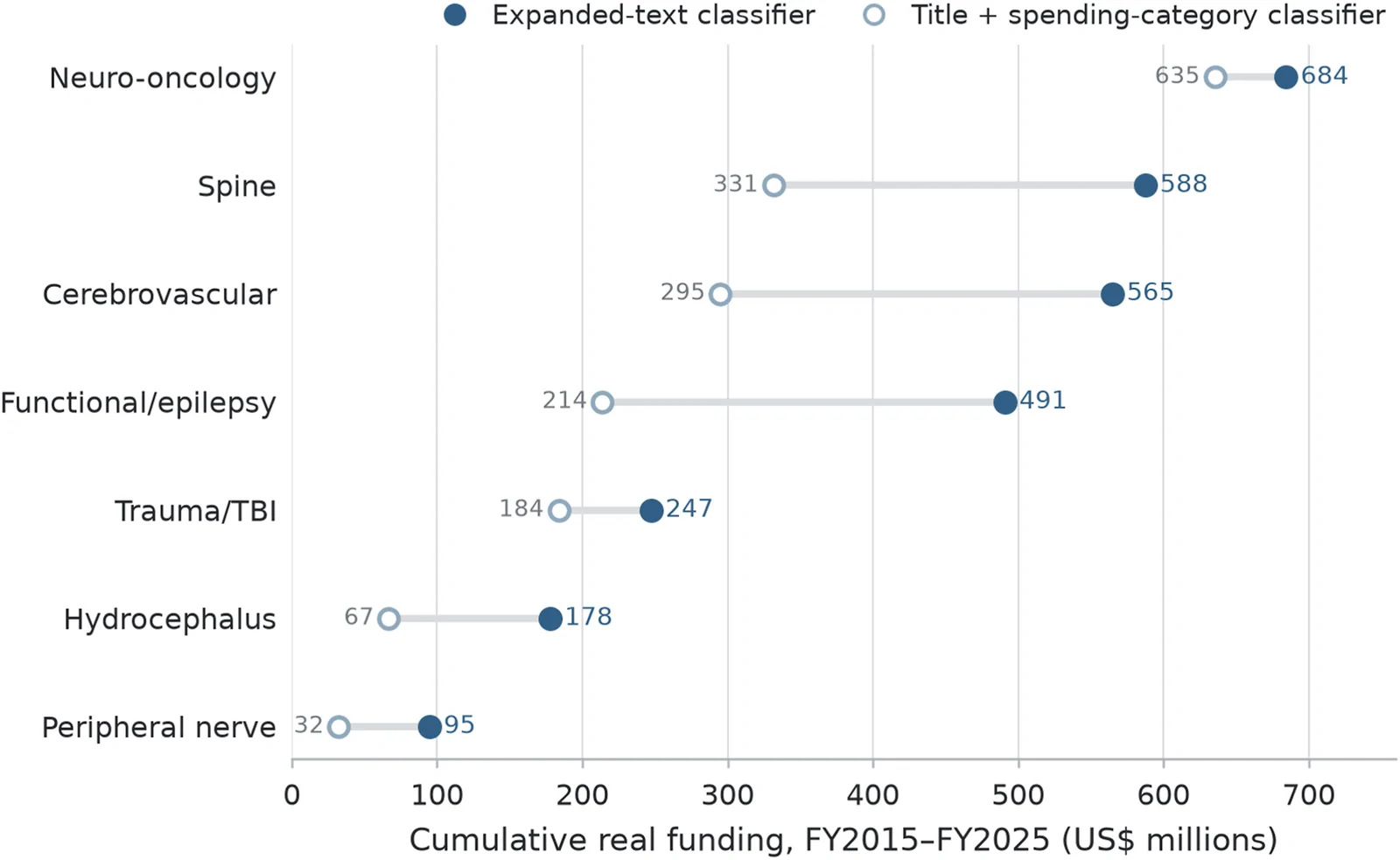
Cumulative keyword-attributed real NIH funding by neurosurgical disease domain, FY2015–FY2025 (constant 2025 US dollars; multi-label keyword screening). Filled circles show the primary expanded-text keyword classifier; open circles show an independent classifier using only the project title and NIH Spending Categorization. Domain amounts are approximate and sum to more than the cohort total because grants matching several domains contribute to each. Source: NIH RePORTER, accessed 8 March 2026.

### 3.9 MeSH retrieval coverage

MeSH-restricted retrieval of neurosurgical publications returned a declining fraction of the keyword-augmented reference set: in general PubMed the ratio fell monotonically from 0.66 (2015) to 0.45 (2023) (Mann–Kendall τ = −1.0), and in the nine-journal subset the MeSH-indexed fraction fell from 0.43 to 0.30 over the same period (τ = −0.78) (Fig. 5). By 2023, a MeSH-restricted search retrieved less than half as many neurosurgical records as a keyword-augmented search in the general query, and less than one-third of the records identified by venue in the journal-anchored analysis. The steepest decline occurred in 2021–2022, temporally overlapping with the NLM's transition to automated MeSH indexing.

**Fig. 5:**
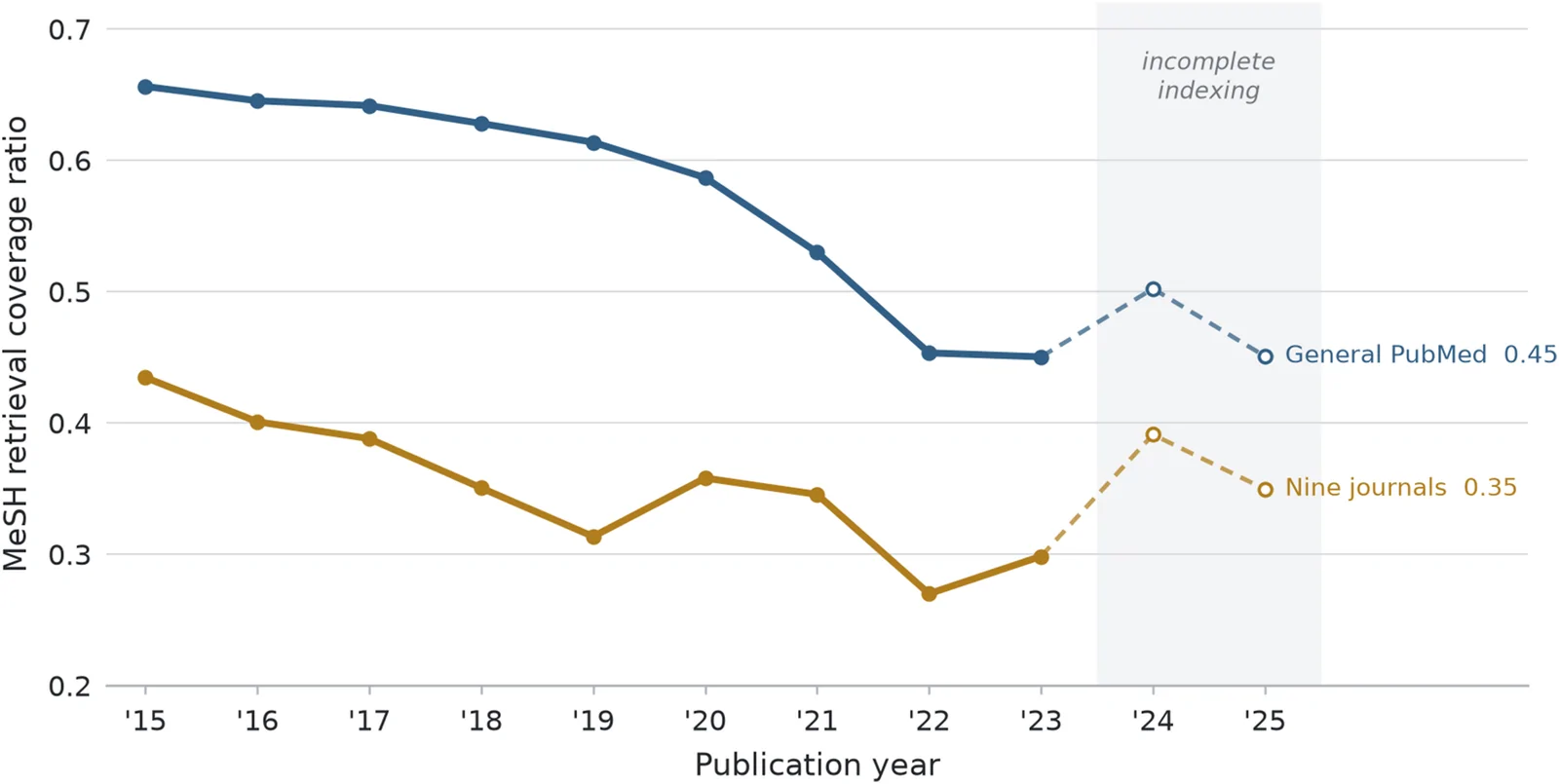
MeSH retrieval coverage of the neurosurgical literature, 2015–2025. The general-PubMed ratio is the count from a MeSH-restricted query divided by the count from a keyword-augmented query; the journal-subset ratio is the count of MeSH-indexed articles divided by all articles in nine core neurosurgery journals. Solid segments (2015–2023) are used for trend testing; dotted segments (2024–2025; shaded) are shown for completeness but are affected by incomplete indexing of recent literature. Source: NCBI E-utilities, queried 20 May 2026.

## 4 Discussion

This analysis of NIH funding to neurosurgery departments yields four findings. First, real-terms funding grew substantially over FY2015–FY2023 but has since fallen below its prior trend. Second, the apparent broadening of the funded base reflects fuller participation of long-established departments rather than new entrants, and concentration has been stable. Third, within public administrative data, independent funding more often followed mentored K awards than fellowships, while the apparent association between institutional T32 grants and R01 capacity was explained by pre-existing departmental research intensity. Fourth, MeSH-restricted retrieval now captures a minority of neurosurgical publications. The funding findings and the retrieval finding are connected by a single theme: both funding attribution and publication retrieval are routine measurements used to judge academic neurosurgery, and both require explicit attention to measurement validity before they are used for evaluation or advocacy.

### 4.1 Relationship to prior work

Our funding analysis updates and extends ReFaey and colleagues, who examined FY1993–FY2017 (ReFaey et al., 2021). The aggregate-funding trend reported here — real-terms growth across most of the window — is concordant with their finding for the overlapping period, and the present study is, in part, an update of that work. Its distinct contribution is a change in analytical question: we adjust for inflation throughout, test the recent trajectory against trend rather than describing it, and examine the award trajectories and institutional dynamics that a descriptive series cannot address. Where ReFaey and colleagues, through manual investigator-level adjudication, documented a declining share of awards held by neurosurgeon-scientists within a growing departmental total (ReFaey et al., 2021), our data cannot identify PI degrees and so cannot speak to that ratio; the two analyses are complementary, and this constraint is fundamental (Section 4.6). Jahangiri and colleagues likewise described long-run NIH funding trends for neurosurgeons, while Lonser and colleagues mapped NINDS opportunities and proposed a coordinated training and career-development framework (Jahangiri et al., 2020; Lonser et al., 2020, 2021). Evidence that Neurosurgery Research and Education Foundation seed awards can convert to later NIH support adds a complementary specialty-level pathway (Smith et al., 2022).

### 4.2 The recent inflection

The downturn after FY2023 is the most time-sensitive finding. The FY2024 and FY2025 values fall below the prediction interval extrapolated from the preceding trend, so the decline is larger than would be expected if the prior log-linear trajectory had simply continued. We are nonetheless cautious: the data are an administrative census and not a probability sample, two post-peak observations cannot establish a durable reversal, and award timing, the FY2025 full-year continuing resolution, and ongoing changes in NIH operations could each contribute. The NIH-wide benchmark provides important context: real RPG obligations and award counts also declined after FY2023, indicating a broader constraint, but the 8.5% departmental decline exceeded the 1.1% RPG funding decline. The 6.5% real decline in the total appropriation was closer in magnitude, although that denominator includes intramural and other spending not directly comparable with departmental awards. Late RePORTER updates may also slightly revise FY2025 totals. The value of the present analysis is that it provides a reproducible baseline and a pre-specified method against which FY2026 and FY2027 data can be assessed as they mature.

### 4.3 Early-career trajectories and training infrastructure

Independent funding followed mentored K awards far more often than fellowships (40.4% versus 8.0%). This is consistent with the design of the two mechanisms — K awards are intended to bridge to independent research funding, whereas fellowships predominantly support predoctoral and early postdoctoral training that need not culminate in a departmental R award — and should not be read as evidence that fellowships are ineffective; fellowship recipients may progress outside neurosurgery departments, outside NIH, beyond the observation window, or into non-PI scientific roles. The observed K-to-independent transition (40.4%) and the median interval of five years are broadly consistent with K-to-R transition rates and timing reported for other surgical specialties (Silvestre et al., 2021; Narahari et al., 2018). The institutional analysis is more cautionary. Departments holding a T32 grant had markedly more R01 investigators in unadjusted comparison, but this difference did not survive adjustment for baseline departmental funding and prior R01-investigator count (adjusted IRR 1.07). The appropriate interpretation is descriptive: T32-bearing departments were already the research-intensive departments, and T32 presence is best read as a marker of established research capacity rather than as an independent driver of it. This complements subspecialty-level concern about the surgeon-scientist pipeline (Pugazenthi et al., 2024; Nguyen et al., 2023). Practical responses supported by the surrounding literature include protected research time, structured grant-writing and senior-investigator mentorship, bridge support across the K-to-R transition, shared methodological cores, early engagement with NINDS programme staff, and specialty seed awards that generate preliminary data (Lonser et al., 2020, 2021; Smith et al., 2022). These strategies are plausible and actionable, but our observational results do not estimate their causal effects.

### 4.4 Institutional concentration

Concentration was stable across the window. The top-ten share remained above half of all funding and the HHI stayed within the conventionally unconcentrated range, indicating that, although a minority of departments hold a disproportionate share, growth was absorbed roughly proportionally rather than flowing disproportionately to the largest departments. The Gini coefficient (0.54–0.58) corroborates this: as an intuitive benchmark only, a value in this range is somewhat higher than the Gini of United States household income (approximately 0.48), though the two are not strictly comparable because one describes funding among a small set of competing departments and the other describes income across a national population. The stability of all three measures, across a decade in which real funding doubled, is the substantive point and contrasts with the increasing concentration described in some national research systems (Aagaard et al., 2020).

### 4.5 MeSH retrieval coverage as a measurement problem

MeSH-restricted retrieval now captures a minority of the neurosurgical literature relative to keyword-augmented and journal-anchored reference sets, and the shortfall has widened over time. This matters because any time series of departmental or specialty output built on MeSH-restricted retrieval will increasingly understate recent output. We are deliberately cautious about mechanism and about the reference sets: keyword-augmented retrieval may itself include false positives, so the ratios quantify retrieval coverage relative to a broader strategy rather than against a true denominator of all neurosurgical publications. The steepest decline, in 2021–2022, is temporally compatible with the NLM's 2022 move to automated MeSH indexing (National Library of Medicine, 2021; Chen et al., 2023), but the present design cannot establish that automated indexing caused the change, which could also reflect indexing policy, evolving PubMed metadata, journal composition, or article-type shifts. The same automated and machine-learning tools that accompanied the change may in time improve coverage and consistency; until coverage is demonstrably restored, temporal bibliometric comparisons in neurosurgery should use keyword-augmented or journal-anchored retrieval rather than MeSH-restricted queries (Chen et al., 2023; Irwin and Rackham, 2017; Hadfield, 2020).

### 4.6 Departmental versus individual funding

The most important limitation is structural. The RePORTER Department field identifies the organisational unit that administers a grant, not the training or degree of the PI. Funding attributed here to “neurosurgery departments” therefore includes awards held by basic scientists and other non-surgeon faculty appointed within those departments, and excludes awards held by neurosurgeons appointed in other departments, such as neurology, oncology, or bioengineering. The focused audit offers partial reassurance rather than complete resolution: 15 of the 20 highest-funded contact PIs were verified neurosurgeons, whereas 5 were other physicians or scientists. Our administrative data contain no systematic PI degree information; consequently we make no claim about funding to neurosurgeons as individuals, and the early-career analysis likewise tracks PIs administratively located in these departments rather than neurosurgeons specifically. Linking RePORTER to a registry such as the National Provider Identifier file would allow surgeon investigators to be distinguished from doctorally trained scientists and is a clear direction for future work (ReFaey et al., 2021). Every result reported here should be read as a property of departments as administrative units. This distinction is especially relevant to current debate about whether the opportunity costs of surgical training, clinical productivity, and protected research time threaten the surgeon-scientist model (Shakil et al., 2026).

### 4.7 Further limitations

Several further limitations apply. The cohort is defined by an exact-string match on the RePORTER Department field and may omit departments recorded under variant labels, neurosurgeons whose awards are administered outside a medical-school neurosurgery department, and standalone clinical groups without matching department metadata. It is restricted to NIH funding; philanthropic, industry, and Department of Defense support are not captured, so total research investment is underestimated and the mechanism mix is NIH-specific (Pugazenthi et al., 2024). Disease-domain classification by keyword screening is imprecise, and the domains are not symmetrical categories; the attributed amounts are approximate and the rank order, though stable across two strategies, should not be finely interpreted. CPI-U is a general deflator; a biomedical-specific index would yield a modestly different magnitude, although the general and biomedical price indices have converged since 2012, so the direction of the conclusions is robust (Lauer et al., 2023). The early-career analysis tracks contact PIs only and does not capture non-contact multiple-PI roles. We did not link grants to publications or to clinical outcomes, so no causal or productivity inference is warranted. A logical next step is a longitudinal linkage of awards to peer-reviewed output, trial leadership, guideline contributions, and patient-relevant translation, with explicit lag periods and field-normalised measures. Finally, FY2025 data, although essentially complete at the query date, may receive minor administrative revision.

### 4.8 Conclusions

NIH funding to neurosurgery departments grew in real terms through FY2023 but has since fallen below its prior trend. The funded base broadened only through fuller participation of established departments, and concentration was stable. Within public administrative data, independent funding more often followed mentored K awards than fellowships, whereas the apparent advantage of institutional T32 grants reflected pre-existing departmental research intensity. MeSH-restricted literature retrieval now captures a minority of neurosurgical publications and should not be used unguarded for temporal bibliometric comparison. Across both the funding and the bibliometric findings, the recurring lesson is that the routine measurements used to evaluate academic neurosurgery — how funding is attributed to departments and how publications are retrieved — must be interpreted with explicit attention to their validity. All results describe departments as administrative units, not individual neurosurgeons, and are reported with a fully reproducible pipeline.

## Ethics approval

This study used publicly available administrative data and aggregate bibliometric counts, did not constitute human-subjects research, and did not require institutional review board approval.

## CRediT author contributions

Aaron Lawson McLean: conceptualisation, methodology, formal analysis, data curation, visualisation, writing – original draft. Julian Kahr: methodology, validation, writing – review and editing. Christian Senft: conceptualisation, supervision, writing – review and editing. All authors approved the final manuscript.

## Data and code availability

The NIH RePORTER export underlying this study is publicly available at https://reporter.nih.gov and can be regenerated from the documented query. Analysis code, aggregate outputs, CPI-U and NIH benchmark values, and PubMed query outputs are provided as an openly available reproducibility package (https://doi.org/10.5281/zenodo.23334479). The public repository does not redistribute the row-level export or applicant-supplied narrative fields.

## Declaration of generative AI use

The authors used a generative artificial intelligence assistant to support code drafting and language editing. All analysis code, statistical outputs, and manuscript content were reviewed and verified by the authors, who take full responsibility for the work.

## Funding

This research received no specific grant from any funding agency in the public, commercial, or not-for-profit sectors.

## Declaration of competing interest

The authors declare that they have no known competing financial interests or personal relationships that could have appeared to influence the work reported in this paper.
