# Supplementary material for: National Institutes of Health funding to neurosurgery departments, FY2015–FY2025: real-terms trends, training infrastructure, and MeSH retrieval coverage
Source: Brain Spine. 2026 Oct 24;6:107486. doi: 10.1016/j.bas.2026.107486 (PMC13734819; doi:10.1016/j.bas.2026.107486)
Supplement: Supplementary file 1 [file mmc1.docx]

**Supplementary Material**

*National Institutes of Health funding to neurosurgery departments, FY2015–FY2025: real-terms trends, training infrastructure, and MeSH retrieval coverage*

Aaron Lawson McLean, Julian Kahr, Christian Senft

**Contents**

• Methods S1. Disease-domain keyword lexicon

• Methods S2. Inflation deflator and NIH appropriation values

• Methods S3. Deduplication procedure

• Methods S4. PI and institutional attribution

• Table S1. PubMed / NCBI E-utilities search strings

• Table S2. Annual institutional concentration metrics

• Table S3. Annual MeSH retrieval counts, 2015–2025

• Table S4. Disease-domain funding and classifier robustness

• Table S5. Deduplication sensitivity: annual funding before and after deduplication

• Table S6. Departments that lost funding between FY2024 and FY2025 (anonymised)

• Table S7. Negative-binomial model of the T32–R01 association

• Table S8. Trend and changepoint test results

• Table S9. NIH-wide post-FY2023 benchmark

• Table S10. Early-career cutoff sensitivity

• Table S11. Manual audit of the 20 highest-funded contact PIs

• Table S12. Grant-mechanism glossary

• Figure S1. Deduplication sensitivity: real funding before and after deduplication

• Figure S2. Departmental funding compared with NIH-wide funding benchmarks

• Figure S3. Lorenz curve of FY2025 departmental funding

**Methods S1. Disease-domain keyword lexicon**

Each grant was screened for seven neurosurgical disease domains by case-insensitive matching of the lexicon below against concatenated text fields (project title, project terms, abstract, NIH Spending Categorization, and Public Health Relevance statement). Multi-word terms were matched as substrings; short abbreviations (italicised) were matched with regular-expression word boundaries. Assignment was multi-label. The domains are not symmetrical categories, and the resulting amounts are described throughout as keyword-attributed disease-domain funding, not as definitive subspecialty totals.

| **Domain** | **Keywords (abbreviations matched on word boundaries)** |
| --- | --- |
| Neuro-oncology | glioma, glioblastoma, brain tumor, brain neoplasm, meningioma, brain cancer, neuro-oncology, medulloblastoma, craniopharyngioma, schwannoma, pituitary, brain metastasis, ependymoma, astrocytoma; GBM |
| Cerebrovascular | aneurysm, cerebrovascular, stroke, subarachnoid hemorrhage, arteriovenous malformation, cavernous malformation, intracranial hemorrhage, cerebral hemorrhage, moyamoya, vasospasm, endovascular, carotid; SAH, AVM, ICH |
| Spine | spinal, spine, lumbar, cervical, disc herniation, scoliosis, vertebral, spinal cord injury, spinal cord, laminectomy, degenerative disc, myelopathy, radiculopathy, intervertebral; SCI |
| Trauma/TBI | traumatic brain injury, craniocerebral trauma, subdural, epidural hematoma, skull fracture, concussion, head injury, blast injury, neurotrauma, decompressive craniectomy; TBI |
| Functional/epilepsy | deep brain stimulation, epilepsy, seizure, functional neurosurgery, movement disorder, parkinson, tremor, dystonia, responsive neurostimulation, vagus nerve stimulation, stereoelectroencephalography; DBS |
| Hydrocephalus | hydrocephalus, ventriculoperitoneal, cerebrospinal fluid, normal pressure hydrocephalus, endoscopic third ventriculostomy; NPH, CSF |
| Peripheral nerve | peripheral nerve, brachial plexus, nerve injury, nerve repair, nerve regeneration, nerve graft, nerve transfer, carpal tunnel, ulnar nerve, median nerve, radial nerve, sciatic nerve, neurofibromatosis, neurofibroma, peripheral neuropathy |

**Methods S2. Inflation deflator and NIH-wide benchmark values**

Nominal award amounts were deflated to constant 2025 US dollars using annual-average CPI-U values (Bureau of Labor Statistics series CUUR0000SA0). The deflator for a year is CPI-U(2025) ÷ CPI-U(year). The 2025 annual average was computed by the Bureau from eleven monthly observations because the October 2025 observation was not collected during the federal shutdown. NIH appropriation values, used in the indexed contextual benchmark in Figure S2 and the approximate departmental budget-share calculation, are the total agency appropriation reported by the NIH Office of Budget and Congressional Research Service report R43341. NIH-wide Research Project Grant obligations and award counts were taken from the NIH Data Book, Research Project Grants, FY2015–FY2025 (https://report.nih.gov/nihdatabook/category/4; accessed 12 October 2026).

| **Fiscal year** | **CPI-U (annual avg.)** | **Deflator to 2025** | **NIH appropriation (US$ billion)** |
| --- | --- | --- | --- |
| 2015 | 237.017 | 1.3583 | 30.3 |
| 2016 | 240.007 | 1.3414 | 32.3 |
| 2017 | 245.120 | 1.3134 | 34.3 |
| 2018 | 251.107 | 1.2821 | 37.3 |
| 2019 | 255.657 | 1.2593 | 39.3 |
| 2020 | 258.811 | 1.2439 | 41.7 |
| 2021 | 270.970 | 1.1881 | 42.9 |
| 2022 | 292.655 | 1.1001 | 45.2 |
| 2023 | 304.702 | 1.0566 | 47.7 |
| 2024 | 313.689 | 1.0263 | 47.1 |
| 2025 | 321.943 | 1.0000 | 47.1 |

**Methods S3. Deduplication procedure**

The raw RePORTER export contained 8672 grant-year rows. 164 Application IDs appeared on more than one row (192 surplus rows). For each Application ID, the row with the largest reported Total Cost was retained. In this cohort all 164 sets of duplicated Application IDs carried identical Total Cost values (none differed in cost completeness), so the retention rule does not alter any grant-year amount; the duplicates are repeated exports of the same award record rather than distinct subprojects or supplements. The deduplicated cohort contained 8480 grant-year records. Deduplication reduced total reported funding from $3.45 billion to $3.32 billion (a 3.9% reduction). Table S5 compares annual funding before and after deduplication; the FY2023 peak and the FY2024–FY2025 decline are present in both series.

**Methods S4. PI and institutional attribution**

PI trajectories were linked by RePORTER Contact PI Person ID across all organizations. Institutional funding, concentration, and persistence were attributed to the Organization Name on each grant-year record; thus, when a PI moved, awards contributed to the recorded institution in the corresponding year. During FY2015–FY2025, 43 of 707 contact PIs were linked to more than one institution. For the focused PI-type audit, the 20 contact PIs with the largest cumulative nominal funding during FY2015–FY2025 were classified from official institutional profiles as a neurosurgeon, non-neurosurgeon physician, or non-physician scientist. The classification and source URL for every audited PI are in Table S11.

**Table S1. PubMed / NCBI E-utilities search strings**

Queries were issued to the NCBI E-utilities esearch endpoint on 20 May 2026. “YYYY” denotes the publication year, iterated over 2015–2025. The keyword-augmented and journal-anchored queries are reference sets for comparison, not gold standards.

**MeSH-restricted query**

("Neurosurgery"[MeSH] OR "Neurosurgical Procedures"[MeSH]) AND "YYYY"[pdat]

**Keyword-augmented query**

("Neurosurgery"[MeSH] OR "Neurosurgical Procedures"[MeSH] OR neurosurg*[tiab] OR craniotomy[tiab] OR craniectomy[tiab] OR laminectomy[tiab] OR discectomy[tiab] OR "spinal fusion"[tiab] OR "deep brain stimulation"[tiab] OR "ventriculoperitoneal shunt"[tiab]) AND "YYYY"[pdat]

**Nine-journal subset query**

("J Neurosurg"[Journal] OR "Neurosurgery"[Journal] OR "World Neurosurg"[Journal] OR "Neurosurg Focus"[Journal] OR "Acta Neurochir (Wien)"[Journal] OR "J Neurosurg Pediatr"[Journal] OR "J Neurosurg Spine"[Journal] OR "Stereotact Funct Neurosurg"[Journal] OR "Neurosurg Rev"[Journal]) AND "YYYY"[pdat]

The MeSH-indexed journal subset adds “AND (“Neurosurgery”[MeSH] OR “Neurosurgical Procedures”[MeSH])” to the nine-journal query.

**Table S2. Annual institutional concentration metrics**

| **FY** | **Funded departments** | **Top-5 share (%)** | **Top-10 share (%)** | **HHI** | **Gini** | **Median funding ($M)** |
| --- | --- | --- | --- | --- | --- | --- |
| 2015 | 45 | 42.6 | 61.3 | 651 | 0.554 | 1.32 |
| 2016 | 47 | 41.6 | 60.0 | 600 | 0.566 | 1.30 |
| 2017 | 48 | 42.1 | 59.8 | 581 | 0.564 | 1.86 |
| 2018 | 46 | 38.7 | 60.8 | 504 | 0.552 | 1.50 |
| 2019 | 52 | 37.6 | 56.8 | 472 | 0.543 | 1.74 |
| 2020 | 54 | 38.0 | 56.2 | 482 | 0.560 | 1.81 |
| 2021 | 54 | 40.0 | 57.9 | 494 | 0.576 | 1.97 |
| 2022 | 52 | 34.8 | 54.5 | 449 | 0.545 | 2.48 |
| 2023 | 55 | 36.8 | 55.7 | 437 | 0.550 | 2.72 |
| 2024 | 59 | 33.9 | 53.5 | 408 | 0.553 | 2.67 |
| 2025 | 54 | 37.8 | 56.3 | 453 | 0.551 | 2.75 |

*HHI, Herfindahl–Hirschman Index (sum of squared percentage shares). Median funding is per funded department, in constant 2025 US dollars. Source: NIH RePORTER, accessed 8 March 2026.*

**Table S3. Annual MeSH retrieval counts, 2015–2025**

| **Year** | **MeSH-restricted** | **Keyword-augmented** | **Ratio** | **Journal: all** | **Journal: MeSH** | **Journal ratio** |
| --- | --- | --- | --- | --- | --- | --- |
| 2015 | 7287 | 11103 | 0.656 | 3766 | 1637 | 0.435 |
| 2016 | 7466 | 11568 | 0.645 | 4594 | 1842 | 0.401 |
| 2017 | 7780 | 12123 | 0.642 | 5179 | 2010 | 0.388 |
| 2018 | 8163 | 12996 | 0.628 | 6253 | 2192 | 0.351 |
| 2019 | 8445 | 13758 | 0.614 | 6841 | 2145 | 0.314 |
| 2020 | 9054 | 15428 | 0.587 | 6549 | 2345 | 0.358 |
| 2021 | 8160 | 15396 | 0.530 | 5509 | 1904 | 0.346 |
| 2022 | 6183 | 13637 | 0.453 | 4952 | 1337 | 0.270 |
| 2023 | 6122 | 13592 | 0.450 | 4714 | 1407 | 0.298 |
| 2024 | 7642 | 15215 | 0.502 | 5583 | 2184 | 0.391 |
| 2025 | 7005 | 15537 | 0.451 | 4473 | 1564 | 0.350 |

*Years 2024–2025 are reported for completeness but were excluded from trend testing because MeSH indexing of recent publications is incomplete. Source: NCBI E-utilities, queried 20 May 2026.*

**Table S4. Disease-domain funding and classifier robustness**

| **Disease domain** | **Primary classifier ($M)** | **Robustness classifier ($M)** | **Grant-years (primary)** |
| --- | --- | --- | --- |
| Neuro-oncology | 684.4 | 635.4 | 1288 |
| Spine | 587.6 | 331.3 | 1163 |
| Cerebrovascular | 564.7 | 294.9 | 1075 |
| Functional/epilepsy | 490.7 | 213.7 | 869 |
| Trauma/TBI | 247.2 | 183.7 | 503 |
| Hydrocephalus | 177.7 | 66.7 | 290 |
| Peripheral nerve | 94.5 | 32.3 | 198 |

*The primary classifier uses the expanded set of RePORTER text fields (project title, terms, abstract, NIH Spending Categorization, and Public Health Relevance statement); the robustness classifier uses only the project title and NIH Spending Categorization. The rank order of domains is identical under the two classifiers (Spearman rho = 1.00); mean per-grant Jaccard agreement of the label sets was 0.72, and 14.6% of grants matched no domain under the primary classifier. Domain amounts are keyword-attributed and approximate, and sum to more than the cohort total because multi-label grants contribute to each domain.*

**Table S5. Deduplication sensitivity**

Annual funding before deduplication (all 8,672 export rows) and after deduplication (8,480 records), in constant 2025 US dollars. Both series peak in FY2023 and decline through FY2025; deduplication shifts the level but not the shape of the trajectory.

| **FY** | **Before dedup., nominal ($M)** | **After dedup., nominal ($M)** | **Before dedup., real ($M)** | **After dedup., real ($M)** |
| --- | --- | --- | --- | --- |
| 2015 | 105.6 | 96.6 | 143.4 | 131.2 |
| 2016 | 127.5 | 111.4 | 171.0 | 149.4 |
| 2017 | 142.5 | 128.3 | 187.2 | 168.5 |
| 2018 | 145.7 | 137.5 | 186.8 | 176.3 |
| 2019 | 171.3 | 165.8 | 215.7 | 208.8 |
| 2020 | 170.2 | 166.5 | 211.7 | 207.1 |
| 2021 | 205.7 | 196.8 | 244.4 | 233.8 |
| 2022 | 241.4 | 231.1 | 265.6 | 254.2 |
| 2023 | 261.7 | 254.1 | 276.5 | 268.5 |
| 2024 | 259.0 | 248.7 | 265.8 | 255.3 |
| 2025 | 254.2 | 245.7 | 254.2 | 245.7 |

*Real compound annual growth FY2015–FY2025 was 5.9% before deduplication and 6.5% after. The real-funding peak fiscal year was FY2023 in both series; the FY2023-to-FY2025 real decline was -8.1% before deduplication and -8.5% after.*

**Table S6. Departments that lost funding between FY2024 and FY2025**

Five departments funded in FY2024 had no funding in FY2025, and no department gained funding over the same interval. Departments are labelled A–E by descending FY2024 funding and are not named. Each held a single grant-year in FY2024.

| **Department** | **Funded years through FY2024** | **FY2024 funding ($)** | **FY2024 grant-years** |
| --- | --- | --- | --- |
| Department A | 16 | $511,734 | 1 |
| Department B | 19 | $498,627 | 1 |
| Department C | 2 | $337,562 | 1 |
| Department D | 21 | $110,001 | 1 |
| Department E | 30 | $49,777 | 1 |

*Four of the five departments had been funded for 16 years or longer, indicating that the FY2024–FY2025 contraction reflects established departments losing a single small award rather than the exit of new entrants.*

**Table S7. Negative-binomial models of the T32–R01 association**

Outcome: count of distinct R01 contact principal investigators per institution, FY2021–FY2025 (n = 87 institutions). Exposure: holding a T32 institutional training grant during FY2010–FY2020. Models were fitted as negative-binomial-family generalised linear models; the negative-binomial family was used in preference to Poisson regression to accommodate overdispersion (the Poisson model's Pearson dispersion statistic was 2.23, exceeding the value of 1 expected under equidispersion).

**T32 incidence rate ratio: unadjusted vs adjusted**

| **Model** | **T32 incidence rate ratio** | **95% confidence interval** | **p-value** |
| --- | --- | --- | --- |
| Unadjusted | 3.52 | 1.69–7.32 | <0.001 |
| Adjusted for baseline funding and baseline R01-PI count | 1.07 | 0.45–2.56 | 0.87 |

**Full adjusted-model coefficients**

| **Term** | **Coefficient** | **exp(coefficient)** | **95% CI** | **p-value** |
| --- | --- | --- | --- | --- |
| Intercept | -0.653 | 0.520 | 0.254–1.067 | 0.075 |
| T32 grant held (FY2010-2020) | 0.070 | 1.073 | 0.449–2.559 | 0.875 |
| Log baseline FY2010-2020 NIH funding | 0.079 | 1.082 | 1.023–1.144 | 0.006 |
| Baseline R01-PI count (FY2010-2020) | 0.129 | 1.138 | 1.070–1.211 | <0.001 |

*For the T32 term, exp(coefficient) is the adjusted incidence rate ratio; for the Intercept it is the modelled rate when all covariates equal zero. Model fit: n = 87 institutions; log-likelihood = -177.1; Akaike information criterion = 362.3; residual deviance = 62.0 on 83 degrees of freedom. The unadjusted T32 association does not persist after adjustment for baseline departmental research intensity: once baseline funding and the baseline R01-PI count are included, those two covariates carry the association and the T32 term is no longer distinguishable from the null. T32-holding departments were already the larger, more research-intensive departments.*

**Table S8. Trend and changepoint test results**

| **Test** | **Statistic** | **Result** |
| --- | --- | --- |
| Mann–Kendall, real funding FY2015–FY2025 | tau = 0.82 | p < 0.001 |
| Mann–Kendall, real funding FY2015–FY2023 | tau = 0.94 | p < 0.001 |
| Mann–Kendall, funded-department count FY2015–FY2025 | tau = 0.71 | p = 0.003 |
| FY2024 observed vs FY2015–FY2023 projection | $255.3M vs $302.1M | 95% PI $271.2–336.6M; below interval |
| FY2025 observed vs FY2015–FY2023 projection | $245.7M vs $329.9M | 95% PI $294.3–369.8M; below interval |
| Mann–Kendall, general MeSH:keyword ratio 2015–2023 | tau = -1.00 | p < 0.001 |
| Mann–Kendall, journal MeSH ratio 2015–2023 | tau = -0.78 | p = 0.005 |

*Prediction intervals are computed on the logarithmic scale and back-transformed; because the data are an administrative census rather than a probability sample, intervals are used descriptively to indicate departure from the extrapolated FY2015–FY2023 trend.*

**Table S9. NIH-wide post-FY2023 benchmark**

All dollar series are expressed in constant 2025 US dollars using the same CPI-U deflator. The series differ in scope and are used for contextual comparison rather than as directly exchangeable denominators.

| **Series** | **FY2023** | **FY2024** | **FY2025** | **FY2023–FY2025 change** |
| --- | --- | --- | --- | --- |
| Neurosurgery-department funding ($B) | 0.268 | 0.255 | 0.246 | -8.5% |
| NIH-wide RPG obligations ($B) | 26.80 | 26.40 | 26.50 | -1.1% |
| Total NIH appropriation ($B) | 50.38 | 48.34 | 47.10 | -6.5% |
| NIH-wide RPG award count | 41597 | 41470 | 39239 | -5.7% |

*RPG, Research Project Grant. Sources: NIH RePORTER (departmental series); NIH Data Book (RPG obligations and counts); NIH Office of Budget and Congressional Research Service R43341 (appropriations).*

**Table S10. Early-career cutoff sensitivity**

The primary FY2019 cutoff provides at least six fiscal years of potential follow-up through FY2025, longer than the observed median five-year transition interval among converters. Adjacent cutoffs yielded similar rates.

| **Latest entry FY** | **Cohort** | **Transitioned** | **Transition rate (%)** | **Median years to R among converters** |
| --- | --- | --- | --- | --- |
| 2017 | 233 | 51 | 21.9 | 6.0 |
| 2018 | 254 | 53 | 20.9 | 6.0 |
| 2019 | 266 | 55 | 20.7 | 5.0 |
| 2020 | 280 | 56 | 20.0 | 5.0 |

**Table S11. Manual audit of the 20 highest-funded contact PIs**

Ranking is based on cumulative nominal funding attributed to each Contact PI Person ID during FY2015–FY2025. PI type was verified against the official institutional profile at the listed URL on 12 October 2026.

| **Rank** | **Contact PI** | **Funding ($M)** | **Classification** | **Official source** |
| --- | --- | --- | --- | --- |
| 1 | SAMPSON, JOHN H. | 38.7 | neurosurgeon | https://neurosurgery.duke.edu/john-h-sampson |
| 2 | HOH, BRIAN LIM | 31.3 | neurosurgeon | https://neuroscience.ufl.edu/profile/hoh-brian/ |
| 3 | LESNIAK, MACIEJ S | 30.3 | neurosurgeon | https://www.feinberg.northwestern.edu/faculty-profiles/az/profile.html?xid=34049 |
| 4 | CHANG, EDWARD | 28.7 | neurosurgeon | https://braintumorcenter.ucsf.edu/people/edward-chang |
| 5 | SOLTESZ, IVAN | 28.1 | non-physician scientist | https://med.stanford.edu/profiles/ivan-soltesz |
| 6 | CHANG, SUSAN M | 26.0 | non-neurosurgeon physician | https://www.ucsfhealth.org/providers/susan-chang |
| 7 | SMITH, DOUGLAS HAMILTON | 25.6 | non-neurosurgeon physician | https://www.med.upenn.edu/apps/faculty/index.php/g20000440/p10366 |
| 8 | BERGER, MITCHEL S. | 25.4 | neurosurgeon | https://braintumorcenter.ucsf.edu/people/mitchel-berger |
| 9 | LANG, FREDERICK F | 25.2 | neurosurgeon | https://faculty.mdanderson.org/profiles/frederick_lang.html |
| 10 | SHETH, SAMEER ANIL | 22.0 | neurosurgeon | https://www.bcm.edu/people-search/sameer-sheth-30585 |
| 11 | MITCHELL, DUANE A. | 21.0 | non-neurosurgeon physician | https://neuroscience.ufl.edu/profile/mitchell-duane/ |
| 12 | LIAU, LINDA M | 20.8 | neurosurgeon | https://www.uclahealth.org/providers/linda-liau |
| 13 | ALVAREZ-BUYLLA, ARTURO | 20.1 | non-physician scientist | https://braintumorcenter.ucsf.edu/people/arturo-alvarez-buylla |
| 14 | BANKIEWICZ, KRYSTOF S | 19.9 | neurosurgeon | https://medicine.osu.edu/find-a-researcher/krzysztof-bankiewicz-100004112 |
| 15 | HENDERSON, JAIMIE M | 18.3 | neurosurgeon | https://profiles.stanford.edu/jaimie-henderson |
| 16 | SCHIFF, STEVEN J | 17.6 | neurosurgeon | https://medicine.yale.edu/profile/steven-schiff/ |
| 17 | POURATIAN, NADER | 15.6 | neurosurgeon | https://profiles.utsouthwestern.edu/profile/205161/nader-pouratian.html |
| 18 | ZIPFEL, GREGORY J | 15.5 | neurosurgeon | https://neurosurgery.wustl.edu/people/gregory-j-zipfel-md/ |
| 19 | GROSS, ROBERT E | 14.2 | neurosurgeon | https://med.emory.edu/departments/neurosurgery/education/academic_tracks/functional_track/components.html |
| 20 | STEINBERG, GARY K | 13.8 | neurosurgeon | https://med.stanford.edu/profiles/gary-steinberg |

*Summary: 15 neurosurgeons, 3 non-neurosurgeon physicians, and 2 non-physician scientists. The audit tests the composition of the highly funded tail; it does not classify all 1,119 contact PIs.*

**Table S12. Grant-mechanism glossary**

| **Code/family** | **Meaning in this analysis** |
| --- | --- |
| F30/F31/F32/F99 | Individual fellowship awards supporting predoctoral, postdoctoral, or transition-stage research training. |
| K series | Mentored career-development awards; K08 and K23 are common clinician-scientist mechanisms. |
| T32 | Institutional National Research Service Award supporting a formal research-training program rather than an individual research project. |
| R01 | Investigator-initiated Research Project Grant; the principal independent project mechanism examined in institutional portfolio analyses. |
| R37 | MERIT award that extends eligible R01 support. |
| RF1/R35/DP1/DP2/DP5 | Independent research mechanisms included with R01/R37 in the PI-trajectory outcome. |
| P01/P30/P50 | Program-project, center-core, and specialized-center grants. |
| U01 | Research Project Cooperative Agreement with substantial NIH involvement. |
| R21 | Exploratory/Developmental Research Grant. |
| UG3/UH3 | Phased exploratory/developmental cooperative-agreement mechanisms. |

**Supplementary figures**

**Figure S1.** Annual funding in constant 2025 US dollars before deduplication (all 8,672 export rows) and after deduplication (8,480 records); the lower strip gives the percentage removed in each fiscal year. The FY2023 peak and subsequent decline are present in both series. Source: NIH RePORTER, accessed 8 March 2026.

**Figure S2.** Inflation-adjusted neurosurgery-department funding, NIH-wide Research Project Grant obligations, and the total NIH appropriation, indexed to FY2015 = 100. The series differ in scope and are shown as a contextual benchmark. Sources: NIH RePORTER; NIH Data Book; NIH Office of Budget and Congressional Research Service R43341.

**Figure S3.** Lorenz curve of NIH funding across the 54 neurosurgery departments funded in FY2025. The diagonal is the line of equality; the shaded area is proportional to the Gini coefficient (0.55). Source: NIH RePORTER, accessed 8 March 2026.
